# Editorial: Omics technologies and fruit postharvest quality

**DOI:** 10.3389/fpls.2024.1404708

**Published:** 2024-04-16

**Authors:** Irene Romero, Ana Margarida Fortes, Fabrizio Costa

**Affiliations:** ^1^ Characterisation, Quality and Safety Department, Institute of Food Science, Technology and Nutrition (ICTAN-CSIC), Madrid, Spain; ^2^ BioISI – Instituto de Biosistemas e Ciências Integrativas, Faculdade de Ciências, Universidade de Lisboa, Lisboa, Portugal; ^3^ Center Agriculture Food Environment (C3A), University of Trento, San Michele all’Adige, Trento, Italy

**Keywords:** fruit, postharvest, quality, omics, chilling injury, ethylene, anthocyanins

## Introduction

The agricultural industry is undergoing a constant change and innovation, driven by the need to meet the growing demand for high-quality fruits in an environment marked by climate change and shifting consumer preferences. In this scenario, postharvest losses pose a major risk to the sustainability of global food systems. Understanding the intricate mechanisms governing fruit quality and postharvest physiology is of paramount importance to ensure sustainable agriculture and consumer satisfaction.

In this Editorial, we present a Research Topic composed by a collection of five original research articles and 1 review, which introduce and discuss recent advances in the understanding of the mechanisms associated with fruit quality and postharvest regulation taking advantage of cutting-edge technologies and innovative approaches. Omics provide valuable insights into the complexity of fruit physiology and metabolism during postharvest treatments. This knowledge will represent a new milestone in modern horticulture, transforming the way we cultivate, harvest, and store high quality fruits under sustainable practices.

## Optimizing parameters of raspberry quality under different cultivation methods

Raspberry (*Rubus idaeus*) stands out for its excellent flavor and high consumer acceptance; therefore, it is crucial to assess how different cultivation methods affect the quality attributes and flavor-related metabolites of this fruit. Fuentealba et al. used untargeted metabolomics to compare the quality of raspberries grown in open fields versus soilless protected cultivation conditions. Significant differences in sugar and amino acid composition were observed, resulting in varied sensory perceptions between the two methods. Raspberries from open fields exhibited higher sugar content, while those from protected soilless culture had higher levels of specific amino acids, suggesting different activation of primary metabolism associated pathways under different cultivation methods. These findings offer insights into raspberry development and postharvest changes, suggesting new practical implications for enhancing fruit quality in alternative cultivation systems.

## Revealing the secrets of anthocyanin accumulation in kiwifruit by integrated omics analyses

Anthocyanins are a crucial component of the pigment in red-fleshed kiwifruit. Ye et al. performed a detailed study on the ‘Jinhongguan’ cultivar of *Actinidia arguta* and unraveled the genes and metabolic pathways responsible for anthocyanin biosynthesis. The combined transcriptome and metabolome analysis by using RNAseq and LC-MS/MS, provided valuable information about the specific types of anthocyanins present in the fruit, as well as the key genes involved in their production. Cyanidin-3-O-galactoside stand out as the main represented anthocyanin while different classes of transcription factors (e.g. MYB) are robust targets to regulate its synthesis. These results not only contribute to our fundamental understanding of fruit biology but also open new opportunities for improving kiwifruit color and quality either by conventional breeding or alternatively by using New Genomic Techniques.

## Addressing chilling injuries in stone and pome fruits during postharvest cold storage

Cold storage is a common method used to prolong the shelf life of harvested fruits, but it can lead to physiological issues like chilling injuries, impacting fruit marketability. Specific apple and pear cultivars, among other fruits, are particularly susceptible to chilling injuries, with superficial scald being a prominent concern due to its economic implications, causing dark lesions on the skin. Peaches and nectarines exhibit, instead, internal symptoms, such as mealiness, rendering affected fruits unsuitable for fresh consumption or further fruit handling. Genetic variation, along with factors such as harvest maturity and storage conditions, influences fruit susceptibility to chilling injuries. Cutting-edge multi-omics approaches, including metabolomics, genomics, and transcriptomics, have provided integrated insights into chilling injury development. By identifying key molecules and pathways involved in cold stress response and fruit metabolism, these approaches reviewed by Rodrigues et al., offer new avenues for enhancing fruit resilience and developing targeted postharvest interventions.

In apple, the work carried out by Vittani et al., disclosed the regulation of superficial scald in “Granny Smith” and “Ladina” apple varieties, assessing two storage strategies: 1-methylcyclopropene (1-MCP) application and controlled atmosphere. Both methods effectively controlled the disorder, revealing two distinct physiological mechanisms triggered by ethylene perception interference or low oxygen concentration. Metabolic and transcriptomic analysis highlighted metabolite reorganization and gene activation associated with disorder prevention. The study emphasized the role of genetic background in superficial scald control, suggesting a cultivar-specific approach for superior postharvest management.

Understanding the molecular mechanisms of chilling injury is essential for developing targeted interventions across fruit varieties. Comparative transcriptomic analyses of peach and nectarine cultivars during postharvest cold storage (Muto et al.) have provided valuable insights into regulatory pathways involved in chilling injury response. While photosynthesis seems to be inhibited, hormonal regulation involving ethylene seems to be activated in both fruits. Despite these shared mechanisms being triggered in both varieties in response to cold storage, cultivar-specific differences highlighted the need for tailored postharvest strategies to mitigate chilling injury risks effectively. 

## Understanding ethylene biosynthesis and signaling in heat-tolerant tomatoes

Tomato cultivation in warm climates presents unique challenges since high temperature affects fruit growth and development. Ethylene is a plant hormone known to play key roles in fruit ripening and postharvest physiology. The ‘Savior’ cultivar has shown heat resistance under various growing conditions. A detailed study on ethylene biosynthesis and signaling in this cultivar (Nguyen et al.) by using targeted molecular and biochemical analyses combined with proteomics revealed significant differences in ethylene metabolism in between winter and summer growing seasons. Ethylene biosynthesis but not signaling account for most of these differences. These findings provide valuable insights for improving heat stress tolerance and fruit quality under challenging environmental conditions.

## Conclusions

A better and advanced understanding in the physiological mechanisms leading to fruit quality are paving the way for more sustainable and resilient agricultural systems and a better control of the postharvest ripening. By harnessing innovative technologies and interdisciplinary approaches, we can address current and future challenges in fruit production, ensuring the world-wide availability and accessibility of high-quality products. A more knowledge-based and science-driven postharvest will also contribute to promoting fruit security by minimizing fruit wastage while enhancing fruit quality. This is an exciting time for the agricultural industry and academia, with the promise that close-future discoveries will enhance our ability to feed a growing population while protecting natural resources.

## Author contributions

IR: Writing – original draft, Writing – review & editing. AF: Writing – review & editing. FC: Writing – review & editing.

